# Designing Neat and Composite Carbon Nanotube Materials by Porosimetric Characterization

**DOI:** 10.1186/s11671-017-2384-2

**Published:** 2017-12-06

**Authors:** Kazufumi Kobashi, Howon Yoon, Seisuke Ata, Takeo Yamada, Don N. Futaba, Kenji Hata

**Affiliations:** 1 National Institute of Advanced Industrial Science and Technology (AIST), CNT-Application Research Center, Tsukuba, Japan; 2 Technology Research Association for Single Wall Carbon Nanotubes (TASC), Tsukuba Central 5, 1-1-1 Higashi, Tsukuba, Ibaraki 305-8565 Japan

**Keywords:** Carbon nanotubes, Agglomerates, Composites, Porosimetry, Pore

## Abstract

We propose a porosimetry-based method to characterize pores formed by carbon nanotubes (CNTs) in the CNT agglomerates for designing neat CNT-based materials and composites. CNT agglomerates contain pores between individual CNTs and/or CNT bundles (micropore < 2 nm, mesopores 2–50 nm, and macropores > 50 nm). We investigated these pores structured by CNTs with different diameters and number of walls, clarifying the broader size distribution and the larger volume with increased diameters and number of walls. Further, we demonstrated that CNT agglomerate structures with different bulk density were distinguished depending on the pore sizes. Our method also revealed that CNT dispersibility in solvent correlated with the pore sizes of CNT agglomerates. By making use of these knowledge on tailorable pores for CNT agglomerates, we successfully found the correlation between electrical conductivity for CNT rubber composites and pore sizes of CNT agglomerates. Therefore, our method can distinguish diverse CNT agglomerate structures and guide pore sizes of CNT agglomerates to give high electrical conductivity of CNT rubber composites.

## Background

A porous medium is a material containing fine pores throughout its matrix. The pores are classified into micropores (< 2 nm), mesopores (2–50 nm), and macropores (> 50 nm) depending on their size by the IUPAC notation. Carbon nanotubes (CNTs), which have drawn much attention as nanoscale fibrous materials with high specific surface areas, are promising as porous materials [1–13]. CNTs possess high aspect ratios (diameter of ~ 1–100 nm, length of several hundred nanometers to several millimeters) and form bundles comprising several to several tens of individual nanotubes by van der Waals force [14, 15]. The CNT bundles become entangled to form CNT agglomerates, thus these CNT structures can construct pores between individual CNTs and/or CNT bundles (micropores, mesopores, and macropores). Based on these porous structures, neat CNT materials exhibit excellent characteristics like high specific surface areas, adsorption capacity, and separation effect; moreover, they can be combined with other materials to form composites. Promising CNT applications are electrode materials, gas and liquid filters, supports for functional microparticles, elastic conductive materials, and structural materials. For these applications, CNTs can be utilized as porous materials in sheet form like Buckypaper [14], bulk form or network structures in matrices such as rubber, resin, and metal, where estimation and control of the pore structures are important. Controlling the pore structures formed by CNTs led to multifunctionality of neat CNT-based materials and composites; however, correlation between the pore structures and their functions has been challenging to investigate.

N_2_ adsorption method has so far been employed to estimate the pores of CNT agglomerates such as Buckypaper [1–7, 9–13, 16]. Both micropores and mesopores, < 50 nm in size, can be measured by this method; however, macropores > 50 nm for CNT agglomerates go out of the range of measurement. Accordingly, we propose a porosimetry capable of measuring macropores > 50 nm to estimate the pore sizes. Porosimetry by mercury intrusion into pores can measure the pore size distribution (pore diameter and volume) over a wide range of several nanometers to several hundred micrometers (mesopores and macropores). The porosimetry utilizes a large surface tension of mercury when the liquid metal intrudes into pores by applying pressure to a porous material. The pore size distribution is then calculated from the pressure and the amount of mercury intruded. Carbon materials have previously been investigated by porosimetry, for carbon fiber strands, graphite, and activated carbon. However, CNT agglomerates have not been comprehensively investigated for the pore sizes which span a few nanometers to several micrometers [16–19].

In order to see its usefulness of porosimetry-based method for CNT agglomerates, we utilized (1) various types of CNT, (2) different forms of CNT agglomerates, (3) CNT dispersions made in different solvents, and (4) different kinds of dispersion methods. These parameters are important to control pore sizes of CNT agglomerates. Firstly, various CNTs (Super Growth single-walled carbon nanotube (SG SWNT), HiPco SWNT, CoMoCAT SWNT, Bayern multi-walled carbon nanotube (MWNT), vapor grown carbon fiber (VGCF)) were dispersed in solvent by a high-pressure jet mill homogenizer. The resulting suspensions were filtered to obtain Buckypapers, then their pores were characterized. The pore sizes of these CNT agglomerates changed depending on the type of CNT (diameter, number of walls), by which we can classify diverse CNTs. Next, we investigated sparsely to densely packed forms of CNT agglomerates and found out that they are distinguishable by the different pore sizes. Furthermore, the correlation between CNT dispersibility in various solvents and pore sizes of CNT agglomerates was demonstrated. When dispersed in *N*,*N*-dimethylformamide (DMF) known to disperse CNTs efficiently, the pore sizes of CNT agglomerates became smaller than those from the poor solvents.

By taking these findings into consideration, we were able to clarify the correlation between electrical conductivity for CNT rubber composites and pore sizes of CNT agglomerates which paves the way to design CNT elastic conductive materials using their pore sizes. We propose this porosimetry-based characterization technology as a standard method to measure pores of CNT agglomerates, which also gives a clear direction to control the pore sizes and design neat CNT-based materials and composites.

## Methods

### CNT Synthesis

SG SWNTs were synthesized in a fully automatic tube furnace by water-assisted chemical vapor deposition using a C_2_H_4_ carbon source on Fe-Ni-Cr alloy metal foils (YEF426) with Fe/Al_2_O_3_ catalyst metal films [8]. The synthesis was done using He with H_2_ as a carrier gas (total flow 1000 sccm) at 1 atm with a controlled amount of water vapor (concentration 100 to 150 ppm). The SWNT growth was carried out at 750 °C with C_2_H_4_ (100 sccm) for 10 min. Height of the SWNT forest synthesized was 100 μm to 1 mm.

### Materials

HiPco SWNT Super Purified, CoMoCAT SWNT CG200, Bayer MWNT Baytubes C70P, and VGCF were purchased from Unidym Inc., Southwest Nanotechnologies, Bayer MaterialScience, and Inc., Showa Denko K. K., respectively. Fluorinated rubber (Daiel-G912) was purchased from Daikin Co.

### CNT Dispersion

CNTs were dispersed at the concentration of 0.03 wt% in solvent by a high-pressure jet-milling homogenizer (60 MPa, 1 pass, nano-jet pal, JN10, Jokoh) except for CNT dispersions to fabricate CNT rubber composite sheets. The solvents used were MIBK, DMF, ethanol, and water. Jet milling exfoliates materials by ejecting suspensions through a nozzle and possesses a significant advantage over other dispersion methods, such as ultrasonication, to suspend long CNTs with minimal shortening effects.

### Preparation of Buckypapers

Filtration of 0.01 wt% CNT dispersions was done by membrane filters with pores of 0.2–0.4 μm. The filter cakes were vacuum dried at 180 °C overnight. Resultant Buckypapers of 4 cm in diameter were ~ 50 μm thick.

### Porosimetry of CNT Agglomerates

Pores (pore diameter and pore volume) of CNT agglomerates were measured by mercury porosimeter (Quantachrome PoreMaster 60). The relationship between applied pressure *P* and the pore diameter into which mercury intrudes *D* is expressed by the Washburn equation: *D* = (− 4γcos*θ*)/*P* where *γ* is the surface tension of Hg (0.48 N m^−1^) and *θ* is the contact angle between mercury and the pore wall (140°) [20]. By monitoring intruded mercury volume against applied pressure, pore size and volume distribution can be obtained based on the Washburn equation. Buckypapers (50–100 mg) were cut into small pieces approximately 5 mm^2^ to load into a porosimeter cell. Regarding SWNT forest and aligned, highly packed SWNTs, small pieces approximately 5 mm^2^ were also loaded into a porosimeter cell of 4 mL by volume.

### Preparation of CNT Rubber Composite Sheets

First, diverse SG SWNT/MIBK dispersions were made at the CNT concentration of 0.125 wt% using three types of dispersion machines: (1) turbulent flow (Nanomizer: 30 MPa 1pass, 100 MPa 1pass, 120 MPa 1pass, in total 3passes, Star Burst Labo: 100 MPa 1pass, 120 MPa 1pass, in total 2passes), (2) cavitation (probe sonicator Vibra-Cell VCX 130: 130 W, 20 kHz, amplitude 100%, 10 min), (3) mechanical force (ball collision-mill Star Burst Mini: ceramic ball, 100 MPa 1pass, 120 MPa 1pass, in total 2passes, bead-mill Dyno-mill: zirconia beads ϕ 0.1 mm, 8 m/s, 120 min, thin-film spin mixer Filmix: 25 m/s, 30 min, paint shaker Toyo Seiki: 750 rpm, 60 min, high shear batch disperser Ultra-turrax: 14,600 rpm, 30 min, rotor-mill Pulverisette 14: 10,000 rpm, 1 min). Second, 10 wt% SG SWNT rubber composite sheets were fabricated by mixing SG SWNT/MIBK dispersion and fluorinated rubber/MIBK solution, then casting in a petri dish, and evaporating the solvent at 25 °C for 16 h, finally drying at 80 °C under vacuum for 6 h. Resultant composite sheets of 4 cm in diameter were ~ 150 μm thick.

### Structural Observation of CNT Agglomerates

Scanning electron microscope FE-SEM S-4800 (Hitachi High-Technologies Co.) was performed to observe the structure of CNT agglomerates. The specimens were made by spin coating the CNT dispersions on Si substrates.

### Electrical Conductivity Measurement of CNT Rubber Composite Sheets

The conductivities of rubber composite sheets were measured with 4-point probe method (MCP-T610, Mitsubishi Chemical Analytech Co., Ltd.). Ten points on a composite sheet were measured to estimate the average value of conductivity and the standard deviation from the surface resistance.

## Results and Discussion

### Various Types of CNT

**Fig. 1:**
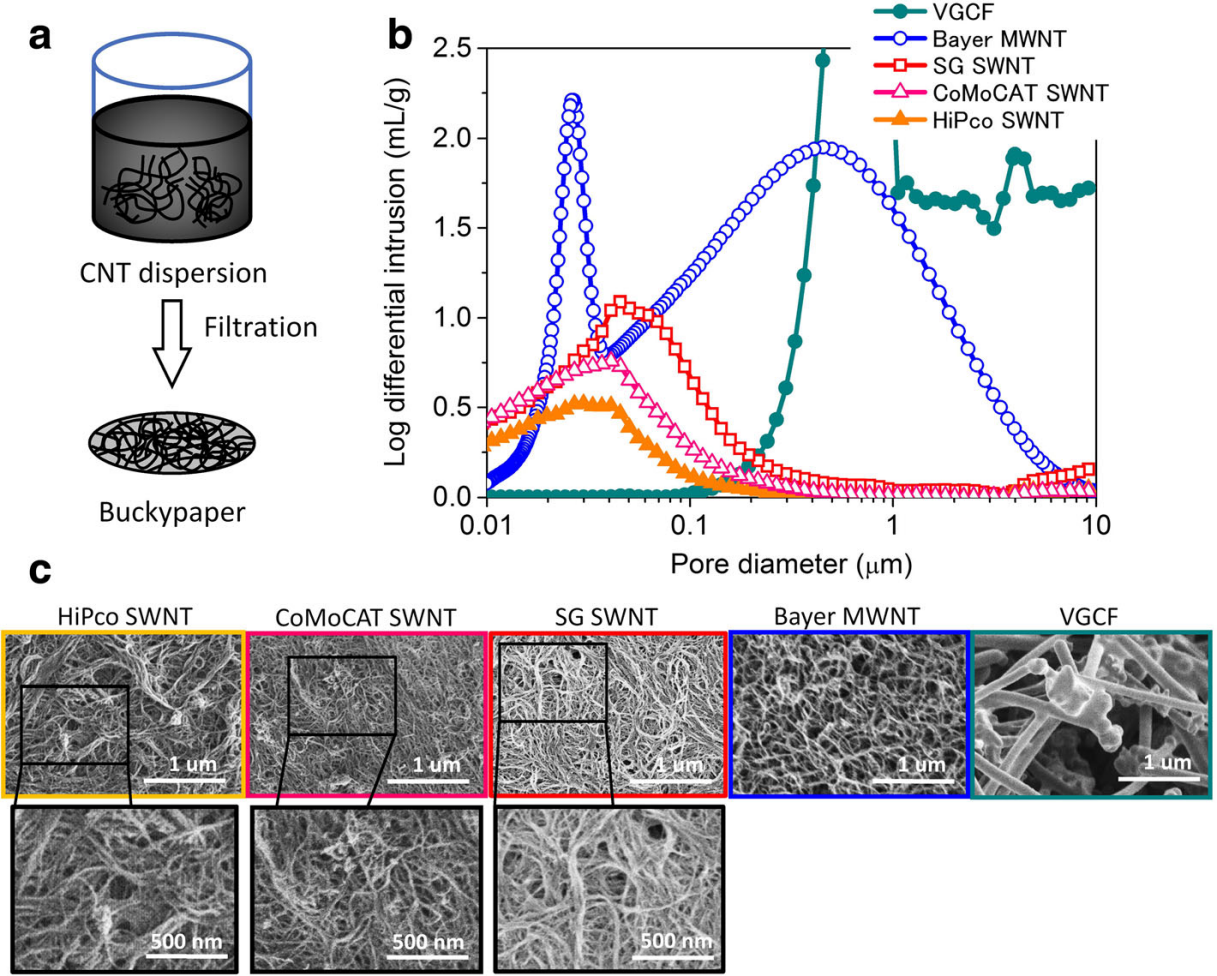
Comparison of pores for various CNTs’ Buckypapers by porosimeter. **a** Schematic for making a Buckypaper, **b** their pore volume (intruded mercury amount) distribution as a function of pore diameter, and **c** SEM images of the various CNTs’ network structures spin-coated on flat surfaces, showing the broadened pore size distribution and increases in the pore volume with increasing CNT diameter (SWNTs to MWNTs)

First, various CNTs were suspended in methyl isobutyl ketone (MIBK) solvent and dispersed through the shear generated by the turbulent flow from a high-pressure jet mill homogenizer to obtain CNT suspensions. The CNT suspensions were filtered to fabricate Buckypapers (Fig. 1a). These Buckypapers were cut into small pieces approximately 5 mm^2^ and loaded into a mercury intrusion porosimeter cell (4 mL). The pores of Buckypapers were then measured using a porosimeter, which encompassed a wide measurement range of 10 nm to 10 μm for mesopores and macropores. The pore volumes (intruded mercury amount: log differential intrusion (mL/g)) were plotted against the pore diameter in Fig. 1b.

Single broad peaks were observed for CNTs having small diameters (CoMoCAT SWNT, diameter 1 ± 0.3 nm, length 1 ± 0.3 μm; HiPco SWNT, diameter ~ 0.8–1.2 nm, length ~ 0.1–1 μm; and SG SWNT, diameter 3 nm, length several hundred micrometers). These peak tops were located around several tens of nanometer pore diameter. On the other hand, broader peaks were observed for CNTs having large diameters (Bayer MWNT, diameter ~ 13 nm, length > 1 μm; VGCF, diameter 150 μm, length 8 μm). The peaks were in the vicinity of 1 μm pore diameter. In case of Bayer MWNT, a sharp rise was observed at 30 nm pore diameter, and it could be attributable to pores between the individual MWNTs [16]. By comparing these various pores, we revealed that the Buckypapers of CNTs with larger diameter and increased number of walls led to the broader pore size distribution and the larger pore volume. Pores > 50 nm in size for CNT agglomerates (macropores) were estimated using a porosimetry, and we demonstrated that pore size distribution changed depending on the type of CNT.

To characterize morphology of these various, porous CNT agglomerates, aliquots of CNT suspensions were spin-coated onto flat substrates, and the scanning electron microscope (SEM) observation showed network structures of entangled CNT agglomerates (Fig. 1c). Fine network structures and pores several tens to 200 nm in size were observed for SWNTs with small diameters. On the other hand, sparse network structures and pores several hundred nanometers to several micrometers in size were observed for MWNTs with large diameters. These observations corresponded with the porosimetry data, which indicated that porosimetry was an efficient method to analyze pores of CNT agglomerates.

### Different Forms of CNT Agglomerates

**Fig. 2:**
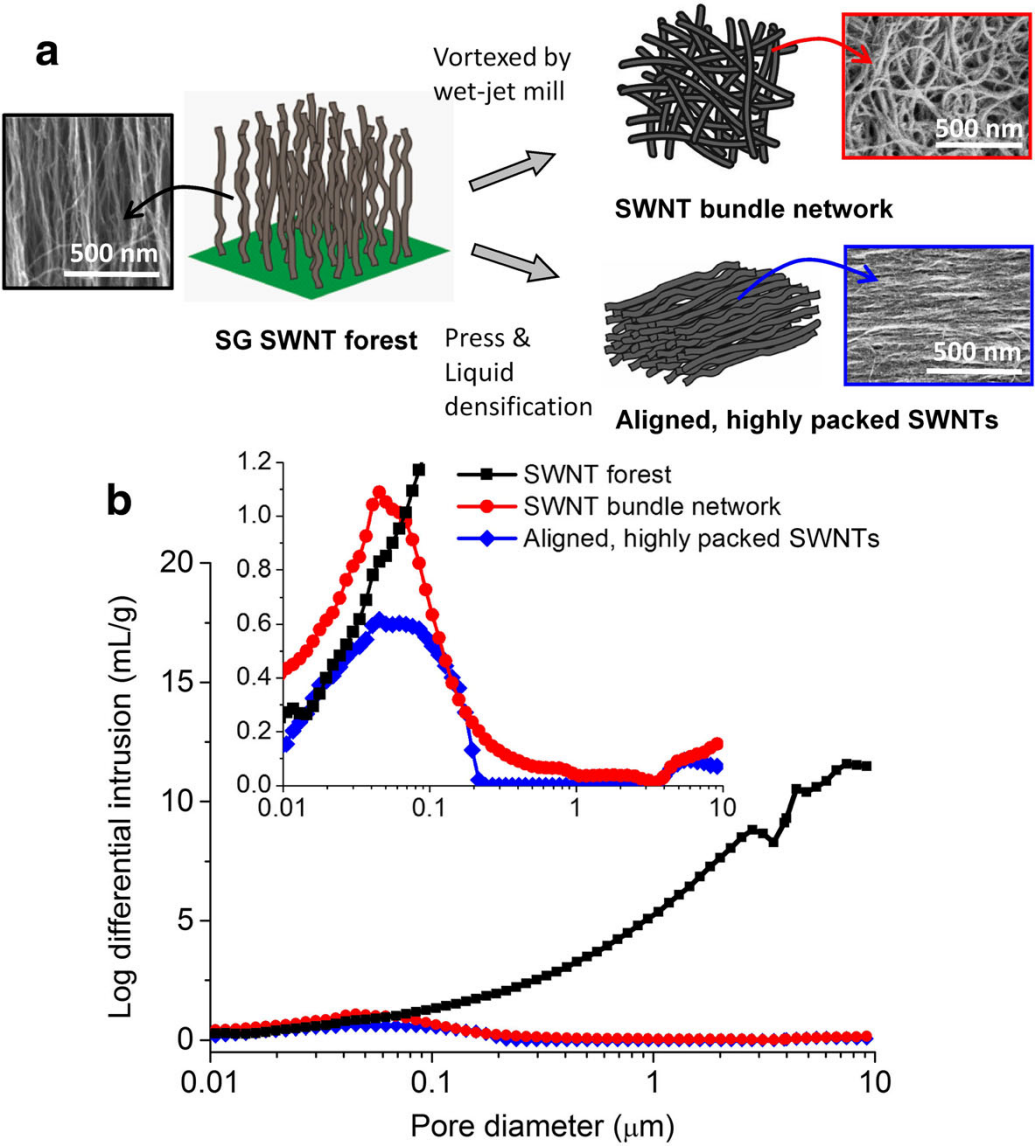
**a** Schematic for conversion of SG SWNT forest into the SWNT bundle network or the aligned, highly packed SWNTs and their SEM images, and **b** comparison of pores for these SWNT structures (Inset: the cutout at log differential intrusion of 0–1.2 mL/g), showing that the sparsely to densely packed SWNT structures can be classified depending on the pores

To distinguish different forms of CNT agglomerates, their bulk density has so far been measured as a macroscopic method; however, the microscopic method has not been reported. Here, we investigated different CNT agglomerate forms from a sparsely packed structure of CNT forest to CNT bundle network with the medium-level packing [21] to aligned, highly packed CNTs [9] (Fig. 2a).

Regarding the three different agglomerate forms comprised of the same CNTs (SG SWNTs), the SEM images were shown in Fig. 2a. First, a sparsely packed structure of SWNT forest was characterized. The SWNTs were grown by the water-assisted chemical vapor deposition (CVD) method (“Super-Growth CVD” method) [8]. In this method, a minute level (~ 150 ppm) of water is inserted into the growth ambient to increase catalyst activity. SWNT forests are very sparse material where SWNTs only occupy < 5% of the volume, the bulk density is low (~ 0.03 g/cm^3^), long and flexible SWNTs are loosely entangled, and they are oriented perpendicularly on the substrate. The SEM observation of SWNT forest showed several tens of nanometers to a few micrometer pores between the oriented SWNTs.

Second, an SWNT bundle network was characterized. This agglomerate form gave the SG SWNTs Buckypaper by filtering the CNT suspension as shown in Fig. 1, which are SWNT agglomerates dispersed from SWNT forests by a high-pressure jet mill homogenizer. The SEM observation showed a network structure of entangled CNT bundles and several tens of nanometer pores (Fig. 2a).

Third, aligned, highly packed SWNTs were fabricated for the porosimetry. When liquids are applied into the sparse SWNT forest and dried, the surface tension of the liquids and the strong van der Waals interactions effectively assemble the nanotubes together to near-ideal graphitic spacing. This packing occurs in two steps: liquid immersion and evaporation, and the nanotubes are drawn together by liquid capillary forces and the forests are densified at the evaporation of liquid [9]. The SEM image of aligned, highly packed SWNTs revealed high-density-oriented CNT agglomerate structures (Fig. 2a). The pores were smaller than those found in both SWNT forests and SWNT bundle network.

Porosimetry results were described as follows for the three different agglomerate forms (Fig. 2b). Pore volume decreased in the order of SWNT forest, SWNT bundle network, and aligned, highly packed SWNTs. This strongly supported the bulk densities of the three different agglomerate forms (0.03, 0.4, 0.6 g/cm^3^) [8, 9] and demonstrated that our method can be used to classify the form of CNT agglomerates. The SWNT bundle network possessed a broader pore size distribution and a larger pore volume than aligned, highly packed SWNTs. As compared with these two CNT agglomerate forms, the pore size distribution for SWNT forest was much broader, and the pore volume was larger. These results corresponded with their pore sizes from SEM observations.

### CNT Dispersions Made in Different Solvents

**Fig. 3:**
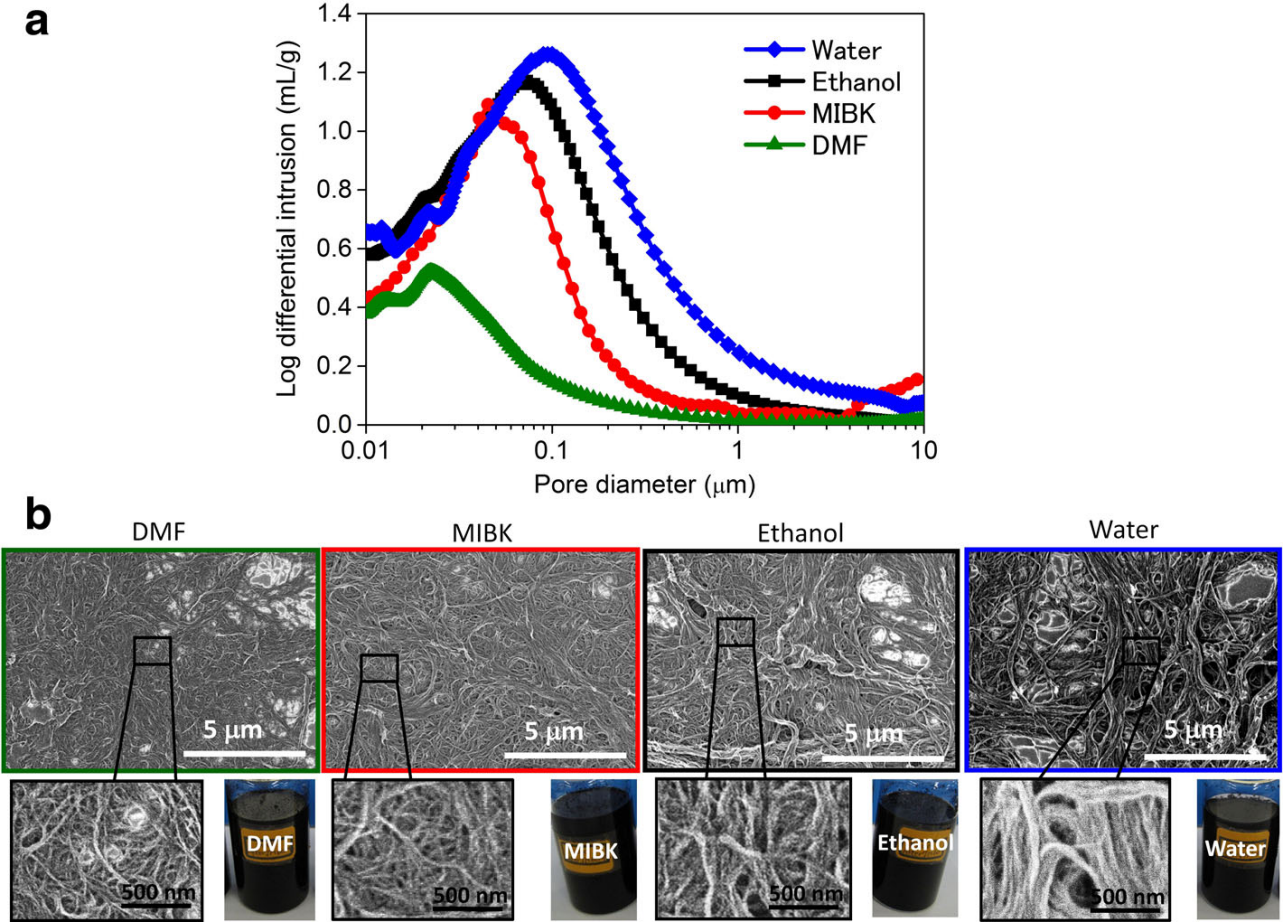
Comparison of pores for SG SWNT Buckypapers made by different solvents. **a** Their pore volume distribution as a function of pore diameter, and **b** SEM images of the various SWNT bundle network structures spin-coated on flat surfaces, photographs of the dispersions, made using dimethylformamide (DMF), methyl isobutyl ketone (MIBK), ethanol and water, showing correlation between CNT dispersibility in solvent and the pore sizes of CNT agglomerates

Furthermore, we report the correlation between CNT dispersibility in solvent and the pore sizes of CNT agglomerates. SG SWNT forests were dispersed in various solvents (DMF, MIBK, ethanol, water) by a high-pressure jet mill homogenizer. All these CNT suspensions were highly stable without precipitation of CNTs (shelf life exceeding 1 year) [21] (Fig. 3). Their Buckypapers were fabricated from the CNT suspensions for the porosimetry. Single broad peaks were observed with the tops around several tens of nanometer pore diameter. Depending on the kind of solvent, the pore diameter with the maximum pore volume (log differential intrusion) increased in the order of DMF, MIBK, ethanol, and water (22, 45, 73, 95 nm). In addition, the pore distribution broadened and the total pore volume increased in the order of DMF, MIBK, ethanol, and water (Fig. 3a).

For SEM observation, aliquots of these CNT suspensions were spin-coated onto flat substrates. Network structures of the CNT agglomerates were observed for all suspensions (Fig. 3b). Regarding CNT dispersibility, the difference in various solvents has been reported [22–26]. DMF is known as a good solvent to more disperse CNTs. Alcohol like ethanol and water are poor solvents for CNTs. MIBK stands at the middle among the good and poor solvents. In this study, the degree of CNT dispersibility changed depending on the solvents; when dispersed in the good solvent, finer CNT bundles were observed and the pore sizes of the CNT agglomerates decreased. These results were in good agreement with the porosimetry measurement.

### Different Kinds of Dispersion Methods

Based on these knowledge for pores of various CNT agglomerates, we investigated correlation between pore sizes of SG CNT Buckypapers and electrical conductivity of the CNT rubber composites. First, to make diverse pore structures formed by SG CNTs, the dispersion in MIBK was carried out by various dispersion methods, which are classified into three types of dispersion mechanisms: (1) turbulent flow (Nanomizer, Star Burst), (2) cavitation (probe sonicator), and (3) mechanical force (ball collision-mill, bead-mill, thin-film spin mixer, paint shaker, high shear batch disperser, rotor-mill) [27].

**Fig. 4:**
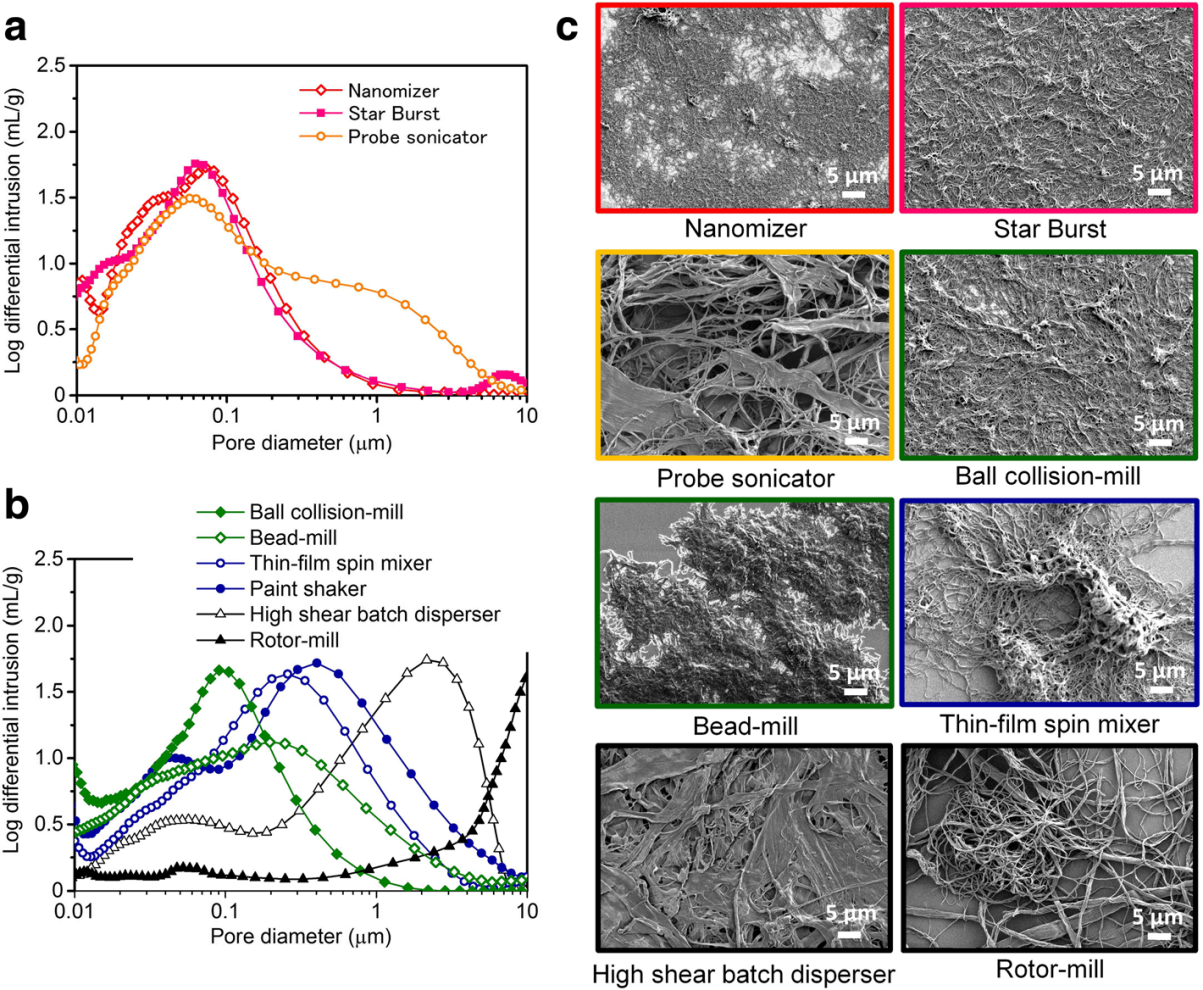
Comparison of pores for SG SWNT Buckypapers made by different dispersion methods. **a**, **b** Their pore volume distribution as a function of pore diameter. **c** SEM images of the various SWNT bundle network structures spin-coated on flat surfaces

These differently dispersed CNTs showed a wide variety of pore size distribution (Fig. 4a, b) and dispersed structures (Fig. 4c). Firstly, turbulent flow-based methods afforded small CNT bundle networks and resulted in fine pores having the pore diameter with the tops around 60–70 nm. Secondly, a cavitation-based method gave a large CNT bundle network with a broad pore size distribution. Thirdly, mechanical force-based methods provided both of small and large CNT bundle networks which possess broad pore size distribution and the pore diameters with a maximum pore volume (log differential intrusion) at greater points of 90 nm to 10 μm than those from turbulent flow-based methods.

**Fig. 5:**
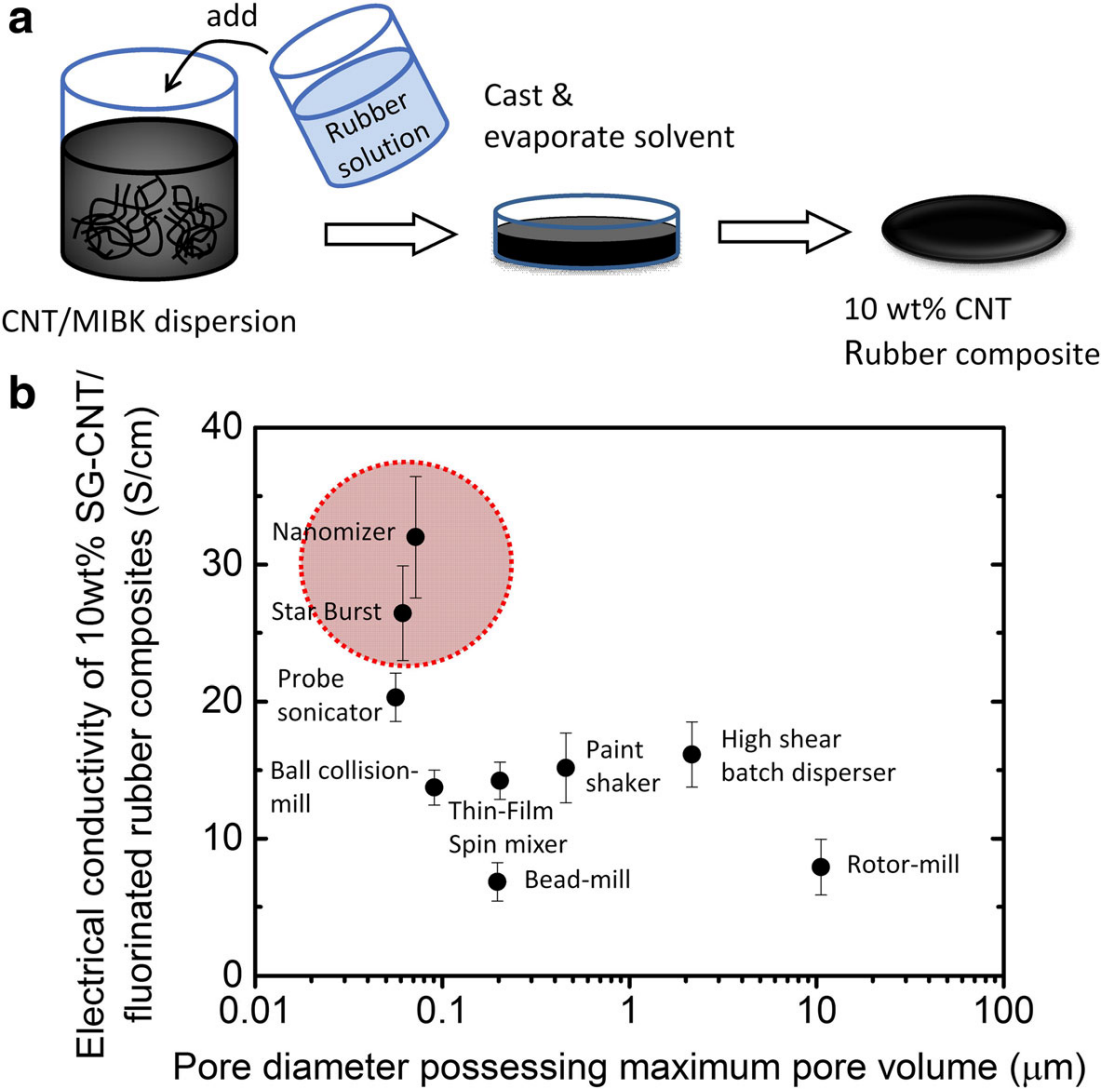
Correlation between pore sizes for SG SWNT Buckypapers and electrical conductivity of SG SWNT/rubber composites. **a** Schematic for making a 10 wt% CNT/rubber composite. **b** Their electrical conductivity as a function of pore diameter possessing maximum pore volume for their Buckypapers

These pores formed by CNTs have a significant influence on performances of neat CNT-based materials like film, sheet, and bulk as well as those of CNT composites. To demonstrate relationship between performance of CNT composites and pore sizes of CNT agglomerates, a CNT rubber composite sheet was chosen as an elastic conductive material. An elastic conductive material, which combines both properties of elasticity and electrical conductivity, is hopeful in the newly emerging field of stretchable electronics. CNT rubber composites have been recently reported to be a synergistic combination of long SWNTs and a fluorinated rubber achieving both electrical conductivity and dynamic durability at high levels [28–30]. To fabricate CNT rubber composites, SG CNT/MIBK dispersion was mixed with fluorinated rubber/MIBK solution. The mixture of SG CNT/rubber/MIBK was cast in petri dish, and the solvent was removed by evaporation and vacuum drying, resulting in the 10 wt% CNT rubber composite sheet (Fig. 5a).

Since the pores formed by CNTs, where rubber was filled, are highly challenging to directly characterize, data of pores formed by differently dispersed CNT agglomerates (Buckypapers, Fig. 4a, b) were used to combine with electrical conductivity of CNT rubber composites. The pore diameters with the tops (pore volume: log differential intrusion) were plotted against electrical conductivity of the CNT rubber composites (Fig. 5b). Turbulent flow-based methods (red-spotted) showed the high electrical conductivity (33, 28 S/cm) of the composite and small pore size diameters with the maximum pore volume (72, 61 nm). A cavitation-based method gave somewhat lower conductivity than those of turbulent-based methods (20 S/cm) and a small pore diameter with the maximum pore volume (56 nm). On the other hand, mechanical force-based methods provided lower conductivity than those of abovementioned methods (< 16 S/cm) and large pore diameters with a maximum pore volume (90 nm to 10 μm).

We found a larger electrical conductivity for CNT rubber composites with smaller pore diameter having a maximum pore volume for Buckypapers. Turbulent flow-based methods have been reported to efficiently exfoliate CNT bundles with a minimum damage to CNTs [27]; the small CNT bundle networks with fine pores (Fig. 4a, c) were beneficial to create high conductivity rubber composites. Although other dispersion methods can also exfoliate CNT bundles, the degree of exfoliation were weaker and the pore sizes were larger (Fig. 4) as compared with turbulent flow-based methods. Additionally, large damages to CNTs in the dispersion processes were detrimental, which led to a low level of conductivity for the rubber composites.

We have characterized diverse pores classified by types of CNT and dispersion parameters. To control these pores of CNT agglomerates, a dispersion method was more influential than a kind of solvent. However, these findings were based on one kind of CNTs, and further investigation with other CNTs would be desirable from an industrial point of view.

## Conclusions

We have developed porosimetry-based characterization method for pores of CNT agglomerates. A conventional N_2_ adsorption method is available for estimating a part (micropores < 2 nm and mesopores 2–50 nm) of pores of CNT agglomerates; however, characterization for the macropores > 50 nm has not been established. Pores for CNT agglomerates (mesopores and macropores) were successfully characterized for CNTs with different diameters and number of walls, and sparsely to densely packed forms of CNT agglomerates. We also revealed that CNT dispersibility in solvent correlated with the pore sizes of CNT agglomerates. This knowledge was utilized to investigate the correlation between electrical conductivity of CNT rubber composites and pore sizes of CNT agglomerates. Therefore, characterization technologies for pores of CNT agglomerates would be a good guide to design neat CNT-based materials and composites.

Although this method employs mercury, which imposes an environmental burden, it enables to estimate pores (mesopores and macropores) for CNT agglomerates. Furthermore, our method is expected as a fundamental technology to characterize pores of CNT agglomerates and will build a firm platform for applications of neat CNT-based materials and composites.
